# 1-Hydroxypyrene Promotes Bladder Cancer Progression and Tumor-Associated Immunosuppression via MIF

**DOI:** 10.3390/ijms27177778

**Published:** 2026-08-30

**Authors:** Yiting Liu, Zongyu Li, Yingchun Kuang, Ke Chen, Lilong Liu

**Affiliations:** 1 Department of Urology, Tongji Hospital, Tongji Medical College, Huazhong University of Science and Technology, Wuhan 430030, China; 2 Institute of Urology, Tongji Hospital, Tongji Medical College, Huazhong University of Science and Technology, Wuhan 430030, China; 3 Hubei Provincial Clinical Research Center for Minimally Invasive Treatment of Urology, Wuhan 430030, China

**Keywords:** 1-hydroxypyrene, bladder cancer, tumor-associated immunosuppression, macrophage migration inhibitory factor

## Abstract

This study examined how 1-hydroxypyrene (1-OHP) affects bladder cancer (BCa) progression and tumor-associated immunosuppression. Public transcriptomic data and target-prediction databases were used to screen for targets related to 1-OHP and BCa. Machine learning was then used to narrow down the candidate genes. Molecular docking and single-cell RNA sequencing were also used to examine the selected targets and related pathways. The role of the main target was tested in vitro. CCK-8, colony formation, EdU, and Transwell assays were used to assess cell growth, migration, and invasion. Protein expression was examined by Western blotting. A tumor cell–CD8^+^ T-cell co-culture system was used to evaluate changes in CD8^+^ T-cell function. We identified 29 targets shared by 1-OHP and BCa. Machine learning further narrowed these targets to six genes, and macrophage migration inhibitory factor (MIF) was chosen for experimental validation. Treatment with 1-OHP increased BCa cell viability, proliferation, migration, and invasion. Silencing MIF partly reduced these effects. 1-OHP also increased MIF and CD74/CD44 expression and enhanced PI3K/AKT/mTOR signaling. These changes were reduced after MIF silencing. In the co-culture system, 1-OHP-treated BCa cells reduced CD8^+^ T-cell proliferation and function. Ki67, GZMB, and IFNG levels were decreased. MIF knockdown partly reversed these changes. Collectively, our findings show that 1-OHP promotes bladder cancer progression and tumor-associated immunosuppression, at least in part, through a MIF-dependent mechanism. By linking environmental exposure to activation of the CD74/CD44–PI3K/AKT/mTOR axis and impaired CD8^+^ T-cell function, our study provides new insight into the molecular basis of environmentally associated bladder carcinogenesis.

## 1. Introduction

Polycyclic aromatic hydrocarbons (PAHs) are persistent environmental pollutants that can be encountered in both environmental and occupational settings [1]. After PAH exposure, 1-hydroxypyrene (1-OHP) is formed as a metabolite and is commonly measured to estimate the level of exposure [1]. As the major terminal metabolite of PAHs produced in the body following inhalation, ingestion, or dermal absorption, urinary 1-OHP stably and quantifiably reflects an individual’s PAH exposure burden [2,3]. Therefore, urinary 1-OHP is widely used for assessing population health risks associated with air pollution, occupational exposure, and lifestyle factors [4,5,6]. A large body of epidemiological evidence has shown that PAH exposure is closely associated with oxidative stress, DNA damage, inflammatory responses, and increased risks of multisystem diseases, all of which underscore the non-negligible potential carcinogenic and pro-carcinogenic effects of PAHs [7,8].

Bladder cancer (BCa) is a common cancer of the urinary system and causes a large number of cancer-related deaths [9,10]. The bladder urothelium is continuously in contact with compounds excreted in urine. This makes it vulnerable to toxic substances from the environment and their biological effects. PAHs and 1-OHP have been studied for their possible roles in BCa development. Population studies have linked higher 1-OHP levels with the risk of bladder cancer, lung cancer, and other urinary tumors. These effects may involve DNA damage, malignant transformation, and changes in cancer-related signaling [7,11,12,13]. Studies in bladder epithelial cells have also shown that PAH metabolites can cause DNA adduct formation, affect DNA repair, and alter the local inflammatory environment [14,15].Cell and animal studies further suggest that PAH exposure can affect cancer stemness, immune escape, and tumor metabolism [1,16,17]. Most studies, however, have examined these effects separately. How PAH or 1-OHP exposure causes molecular changes in bladder cancer is still not fully understood.

Here, we integrated computational approaches and experimental tests to identify key mediators linking 1-OHP exposure to BCa progression and tumor-associated immunosuppression. Specifically, we combined public transcriptomic data, target-prediction analysis, machine-learning methods, molecular docking, and single-cell transcriptomic analysis to prioritize candidate mediators. Among the identified candidates, macrophage migration inhibitory factor (MIF) was selected for further investigation because of its established roles in tumor progression and immune regulation. Through in vitro functional assays and tumor cell–CD8^+^ T-cell co-culture experiments, we demonstrate that 1-OHP promotes malignant phenotypes and tumor-associated immunosuppression, at least in part, through a MIF-dependent mechanism involving CD74/CD44-associated PI3K/AKT/mTOR signaling. These results help explain the mechanisms underlying environmentally associated bladder carcinogenesis.

## 2. Results

### 2.1. Integrated Bioinformatics Identifies Candidate Targets of 1-OHP in Bladder Cancer

**Figure 1:**
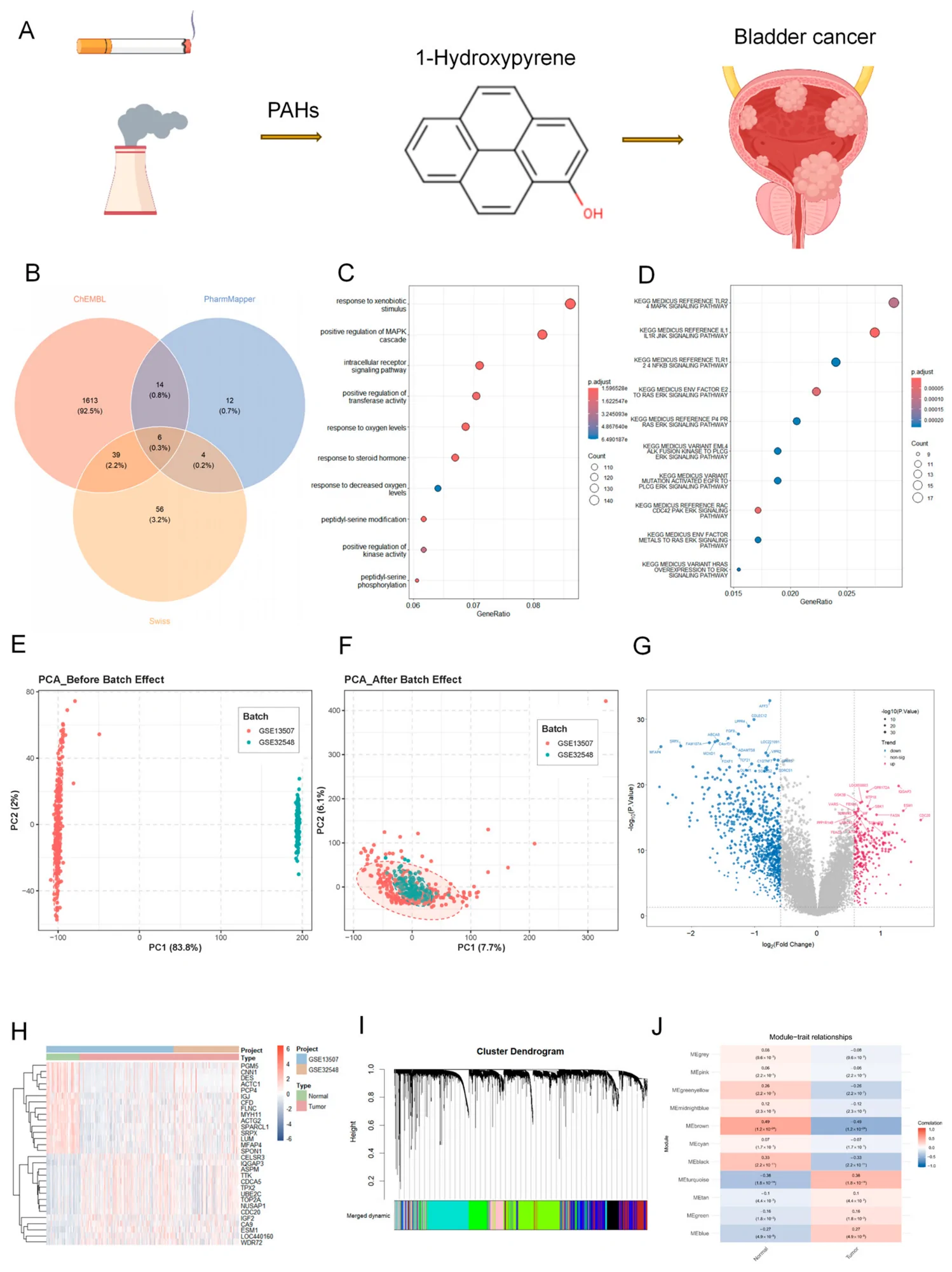
Identification of 1-OHP targets and BCa-related genes. (**A**) Workflow for linking PAHs, 1-OHP, and BCa. (**B**) Candidate 1-OHP targets collected from ChEMBL, PharmMapper, and SwissTargetPrediction. (**C**,**D**) GO terms and KEGG pathways associated with the predicted 1-OHP targets. (**E**,**F**) PCA plots before and after batch correction of the BCa datasets. (**G**,**H**) Volcano plot and heatmap of DEGs. (**I**,**J**) WGCNA clustering, module assignment, and module–trait correlations.

**Figure 2:**
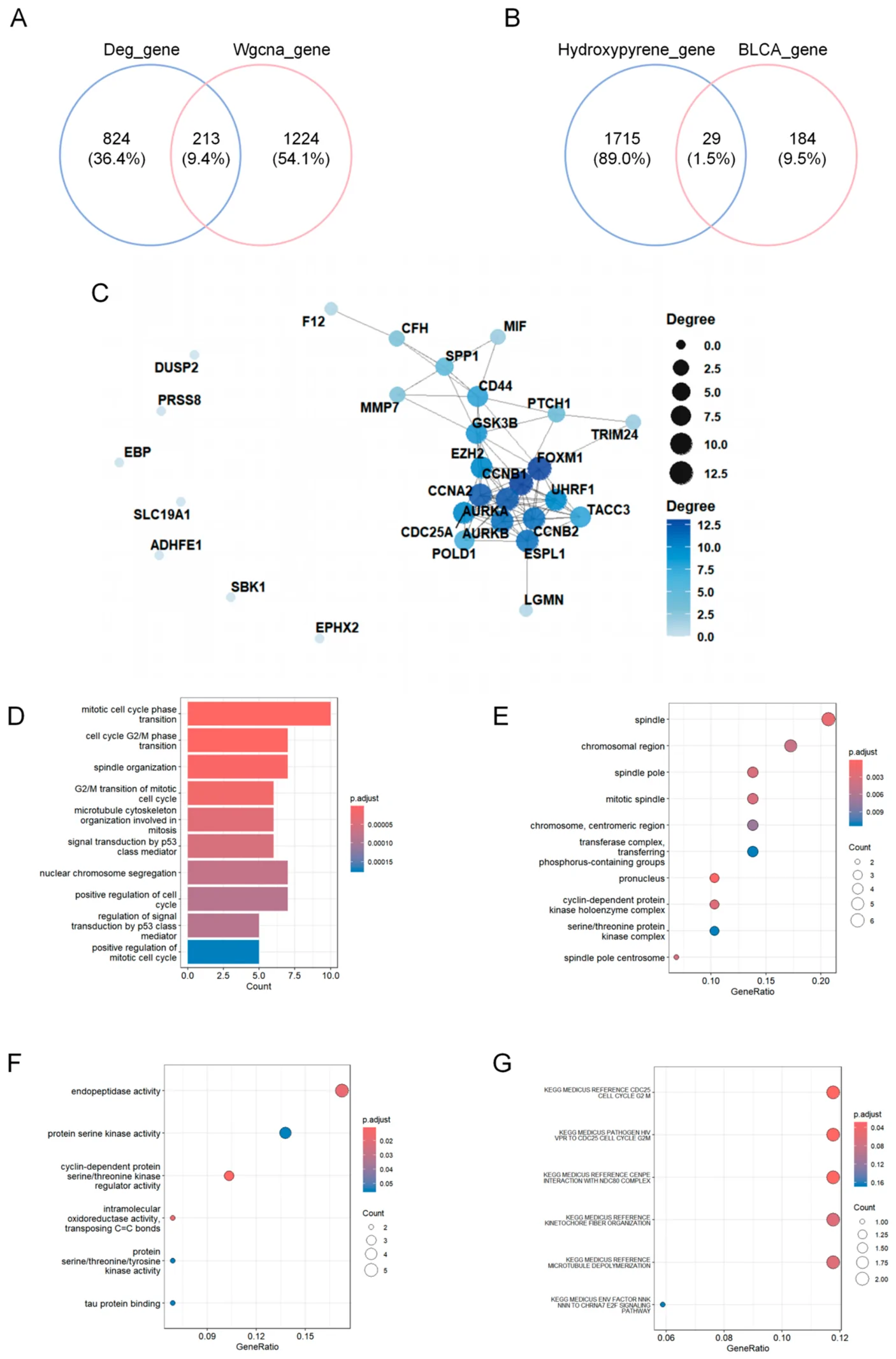
Identification and functional annotation of candidate genes linking 1-OHP to BCa. (**A**) Shared genes between DEGs and WGCNA hub genes. (**B**) Common genes between BCa-related candidates and predicted 1-OHP targets. (**C**) PPI network of the shared candidates. (**D**–**F**) GO results for biological process (BP), cellular component (CC), and molecular function (MF). (**G**) KEGG pathways associated with the candidate genes.

The molecular structure of 1-OHP was obtained from PubChem (Figure 1A). ChEMBL, PharmMapper, and SwissTargetPrediction yielded 1744 unique potential targets after duplicate removal (Figure 1B). GO analysis linked these targets to xenobiotic response, MAPK cascade regulation, intracellular receptor signaling, and oxidative stress response (Figure 1C). KEGG analysis indicated several cancer-related pathways, including RAS, MAPK, and PI3K-AKT signaling (Figure 1D). GSE13507 and GSE32548 were combined and normalized for further analysis. PCA showed better separation after normalization (Figure 1E,F). We identified 1037 DEGs in BCa (Figure 1G,H). WGCNA was then used to examine gene modules related to BCa. A soft-thresholding power of β = 6 was used, and eight co-expression modules were obtained (Figure 1I). The turquoise module had the strongest positive correlation with tumor samples and a negative correlation with normal tissues (Figure 1J). Genes from the turquoise module were compared with the DEGs, resulting in 213 BCa-related candidates (Figure 2A). Comparison of these genes with the predicted 1-OHP targets gave 29 shared genes (Figure 2B). Their interactions are shown in the PPI network (Figure 2C). GO analysis linked these genes mainly to mitotic cell cycle progression, G2/M transition, spindle organization, microtubule dynamics, and p53-mediated signaling (Figure 2D). For cellular components, the main terms included the spindle apparatus, centromeric chromosome regions, and cyclin-dependent kinase complexes (Figure 2E). Molecular function terms were mainly related to serine/threonine kinase activity and cyclin-dependent kinase regulation (Figure 2F). KEGG analysis also linked these genes to G2/M transition, kinetochore organization, microtubule regulation, and E2F signaling (Figure 2G). These results narrowed the initial 1-OHP target set to genes related to cell-cycle control in BCa.

### 2.2. Machine Learning and Molecular Docking Prioritize MIF as a Potential Target of 1-OHP

**Figure 3:**
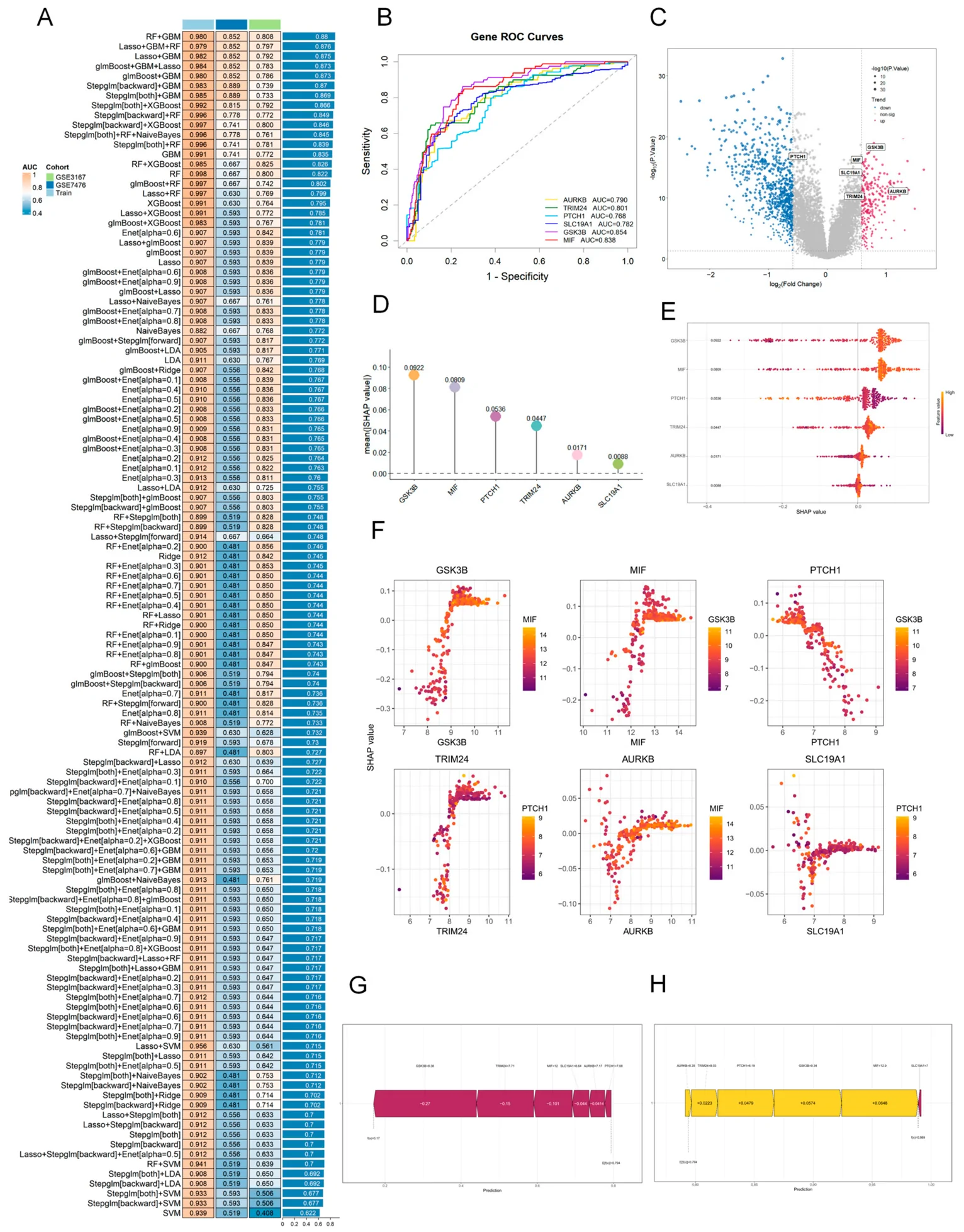
Machine learning-based identification and interpretation of core genes associated with 1-OHP-related BCa. (**A**) Performance of the 113 prediction models. (**B**) ROC curves for the selected models. (**C**) Expression profiles of the six selected genes in BCa. (**D**) SHAP summary plot for gene importance. (**E**) SHAP distribution for the six genes. (**F**) SHAP dependence plots for the relationship between gene expression and model output. (**G**,**H**) SHAP force plots for individual prediction examples.

**Figure 4:**
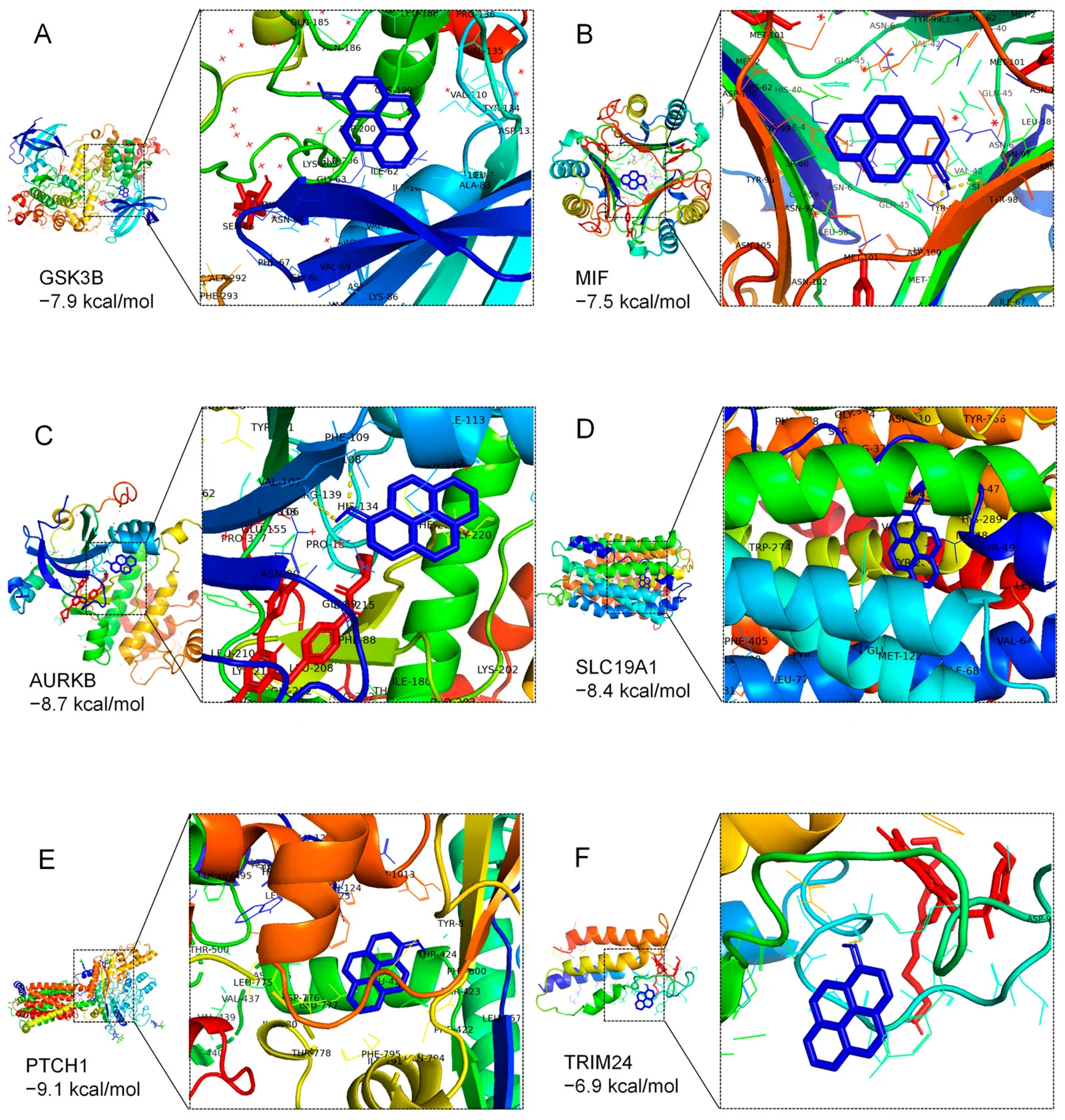
Molecular docking analysis of 1-OHP with the six core target proteins. Representative docking poses of 1-OHP with (**A**) GSK3B, (**B**) MIF, (**C**) AURKB, (**D**) SLC19A1, (**E**) PTCH1, and (**F**) TRIM24. The corresponding binding energies are shown in each panel.

The 29 candidate genes were further examined with 113 prediction models. The RF + GBM model had the best overall performance in both the training and validation datasets (Figure 3A). Six genes were consistently ranked among the main contributors: GSK3B, MIF, PTCH1, TRIM24, AURKB, and SLC19A1. ROC analysis supported the predictive value of these genes, with AUC values above 0.75 (Figure 3B). Their expression patterns in BCa are shown in Figure 3C. SHAP analysis ranked GSK3B (SHAP = 0.0922) and MIF (SHAP = 0.0809) as the two strongest contributors to model prediction (Figure 3D,E). The dependence plots showed different patterns among the six genes (Figure 3F). MIF showed a threshold-like pattern, with SHAP values changing from negative to positive when expression reached about 12. PTCH1 showed an inverse pattern, with higher expression linked to more negative SHAP values. GSK3B and AURKB showed stronger positive effects as their expression increased. TRIM24 showed a biphasic pattern, while SLC19A1 showed a shallow U-shaped pattern. SHAP force plots also showed differences between individual samples (Figure 3G,H). In a low-risk example, GSK3B, TRIM24, MIF, SLC19A1, and AURKB moved the prediction below the baseline. In a high-risk example, TRIM24, MIF, GSK3B, and MIF-related features moved the prediction above the expected baseline. The six proteins were then docked with 1-OHP to assess their possible binding (Figure 4A–F). Binding energies ranged from −6.9 to −9.1 kcal/mol. All six values were below −5.0 kcal/mol, indicating favorable predicted binding. The docking poses showed that 1-OHP could fit into the predicted binding pockets of these proteins. Together, the machine-learning and docking results supported these six genes as potential targets related to 1-OHP in BCa.

### 2.3. Single-Cell Analysis Reveals That MIF^+^ Tumor Epithelial Cells Enhance Immune Interactions and Exhibit Malignant Features

**Figure 5:**
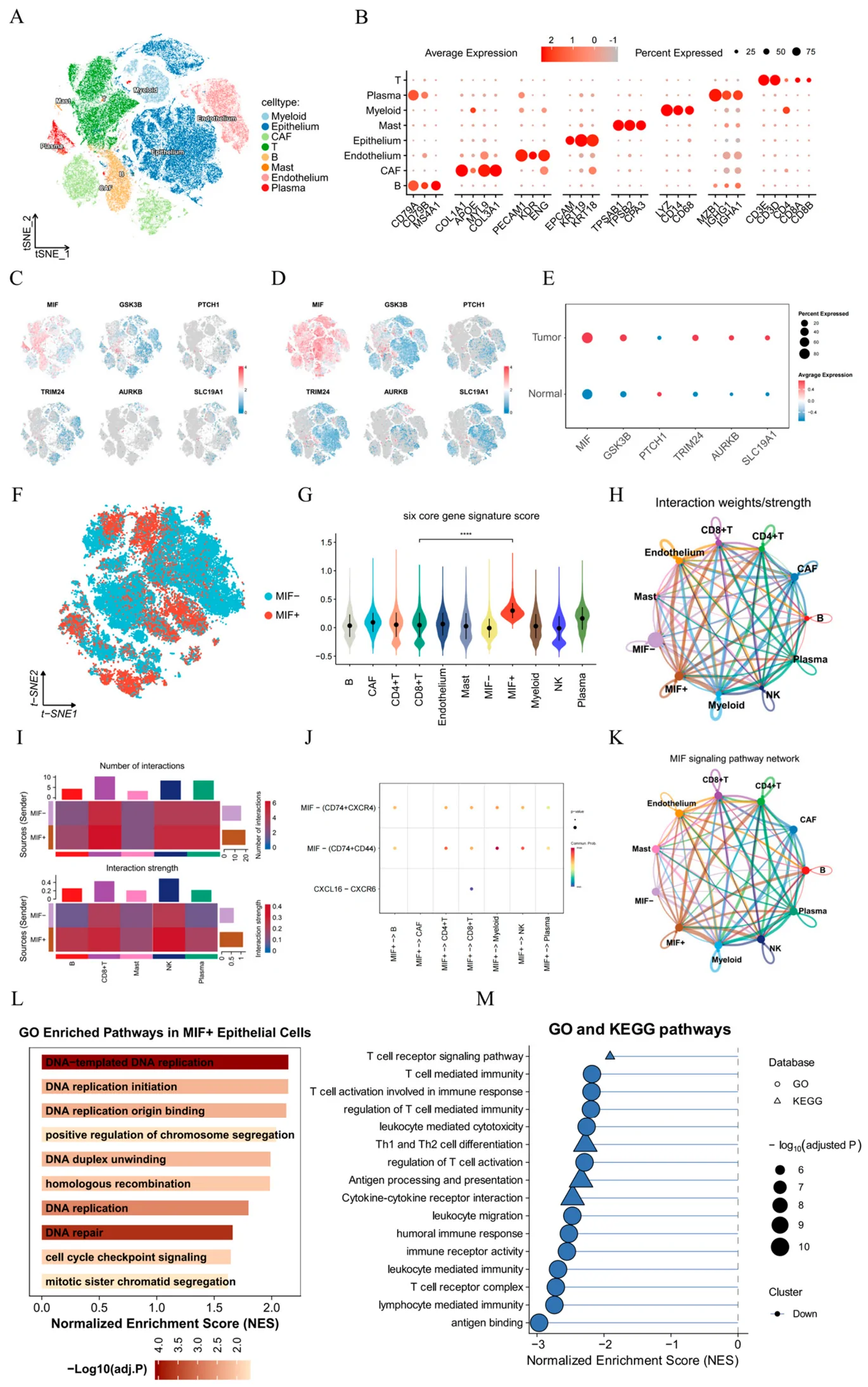
Single-cell analysis reveals that MIF^+^ tumor epithelial cells enhance immune interactions and exhibit malignant features. (**A**) Distribution of 127,242 cells from the integrated GSE222315 and HRA002012 datasets. (**B**) Eight major cell populations defined by canonical markers. (**C**) Expression of the six selected genes across cell types in normal samples. (**D**) Expression of the six selected genes across cell types in tumor samples. (**E**) Comparative expression of the six core genes between normal and tumor tissues. (**F**) Stratification of tumor epithelial cells into MIF^−^Epi and MIF^+^Epi subpopulations based on MIF expression. (**G**) Comparison of the six-gene signature scores between MIF^−^Epi and MIF^+^Epi. The boxplot illustrates the difference in signature scores between the two epithelial subsets, with MIF^+^Epi showing significantly higher scores than MIF^−^Epi. (**H**,**I**) Cell–cell communication analysis comparing MIF^−^Epi and MIF^+^Epi, showing differences in the number and strength of interactions between these epithelial subsets and various immune cell types. (**J**,**K**) Communication probability and significance of MIF-related signaling pathways and their ligand–receptor pairs between MIF^−^Epi and MIF^+^Epi. (**L**) GO and KEGG enrichment analysis of genes upregulated in MIF^+^Epi, indicating the major biological processes and pathways involved. (**M**) Association between MIF expression levels and enrichment of immune-related pathways in the TCGA-BLCA cohort, based on stratification by MIF expression.**** *p* < 0.0001.

After quality control, 127,242 cells from GSE222315 and HRA002012 were included in the single-cell analysis. Eight major cell populations were identified using canonical marker genes (Figure 5A,B). The six core genes showed different expression patterns across these cell types (Figure 5C,D). MIF was the only gene with broad expression across multiple cell types and was higher in tumor tissues than in normal tissues (Figure 5E). This difference was most evident in epithelial cells. The other five genes showed little difference between tumor and normal samples. Epithelial cells were divided into MIF^−^Epi and MIF^+^Epi groups according to MIF expression (Figure 5F). The six-gene signature score was higher in MIF^+^Epi cells (Figure 5G). CellChat analysis was used to examine communication between epithelial and immune cells. MIF^+^Epi cells showed stronger and broader interactions than MIF^−^Epi cells. These interactions were prominent with CD8^+^ T cells and were also seen with CD4^+^ T cells, B cells, NK cells, and myeloid cells (Figure 5H,I). Ligand–receptor analysis showed higher activity of MIF-CD74-CXCR4 and MIF-CD74-CD44 signaling in the MIF^+^Epi group (Figure 5J,K). GO and KEGG analysis linked MIF^+^Epi cells to cell proliferation, DNA replication, DNA repair, and chromosome segregation (Figure 5L). TCGA-BLCA samples were also divided according to median MIF expression. Tumors with high MIF expression had lower activity of immune-related pathways, including T-cell activation, TCR signaling, and T-cell-mediated immune responses (Figure 5M).

### 2.4. 1-OHP Promotes Bladder Cancer Cell Growth and Increases MIF Expression

**Figure 6:**
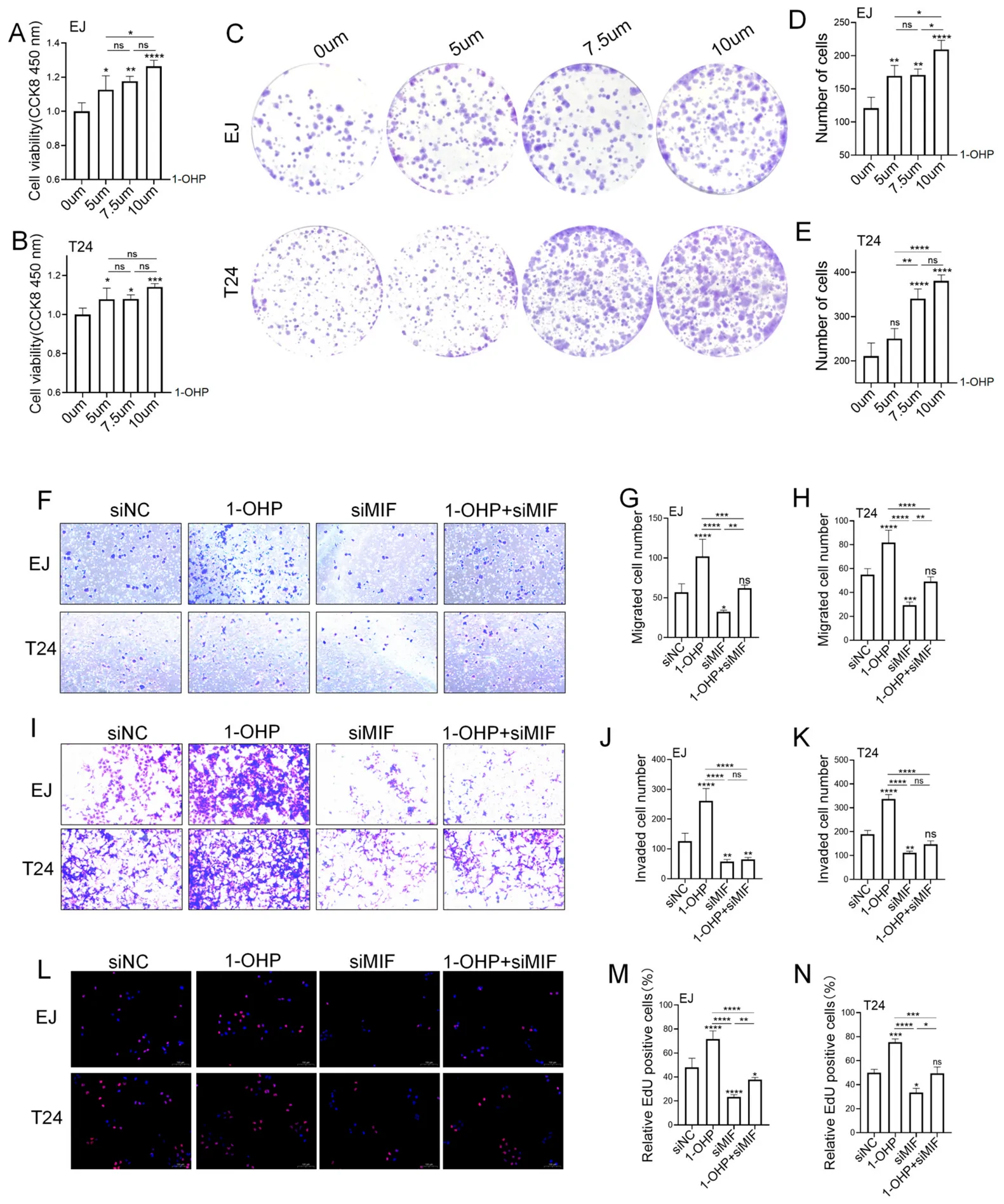
1-OHP promotes malignant phenotypes of BCa cells in a MIF-dependent manner. (**A**,**B**) CCK-8 assays showing the effects of different concentrations of 1-OHP on the viability of EJ and T24 cells. (**C**–**E**) Colony formation assays showing that 1-OHP enhanced the clonogenic ability of EJ and T24 cells (whole-well images). (**F**–**H**) Transwell migration assays showing that MIF silencing attenuated 1-OHP-induced migration of EJ and T24 cells (10× objective). (**I**–**K**) Transwell invasion assays showing that MIF silencing attenuated 1-OHP-induced invasion of EJ and T24 cells (10× objective). (**L**–**N**) EdU assays showing that MIF knockdown partially reversed the promotive effect of 1-OHP on cell proliferation (Scale bar = 100μm.). Data are presented as mean ± SD. ns, not significant; * *p* < 0.05, ** *p* < 0.01, *** *p* < 0.001, **** *p* < 0.0001.

CCK-8 assays were used to examine the effect of 1-OHP (0–10 μM) on BCa cell viability. 1-OHP increased the viability of EJ and T24 cells, with a stronger effect at higher concentrations (Figure 6A,B). Colony formation also increased after 1-OHP treatment in both cell lines (Figure 6C–E). EdU analysis gave a similar result, showing more proliferating cells after treatment (Figure 6F). These results show that 1-OHP promotes BCa cell growth in vitro. Western blotting showed higher MIF protein levels in EJ and T24 cells after 1-OHP treatment than in vehicle-treated controls (Figure S1A,B). Three siRNAs targeting MIF (siMIF-1/-2/-3) were then tested in both cell lines. All three reduced MIF protein levels compared with the siNC group (Figure S1C,D). These results confirmed effective MIF knockdown and allowed further analysis of its role in the effects of 1-OHP.

### 2.5. MIF Mediates the Pro-Tumor Effects of 1-OHP in Bladder Cancer Cells

The role of MIF in the effects of 1-OHP was tested by MIF knockdown. 1-OHP increased the migration and invasion of EJ and T24 cells. These effects were reduced when MIF was silenced (Figure 6F–K). EdU staining showed the same pattern. More EdU-positive cells were detected after 1-OHP treatment, while MIF knockdown reduced this increase (Figure 6L–N). These data show that MIF is involved in the effects of 1-OHP on BCa cell proliferation, migration, and invasion.

### 2.6. MIF Mediates 1-OHP-Induced Immune Suppression and PI3K/AKT/mTOR Activation

**Figure 7:**
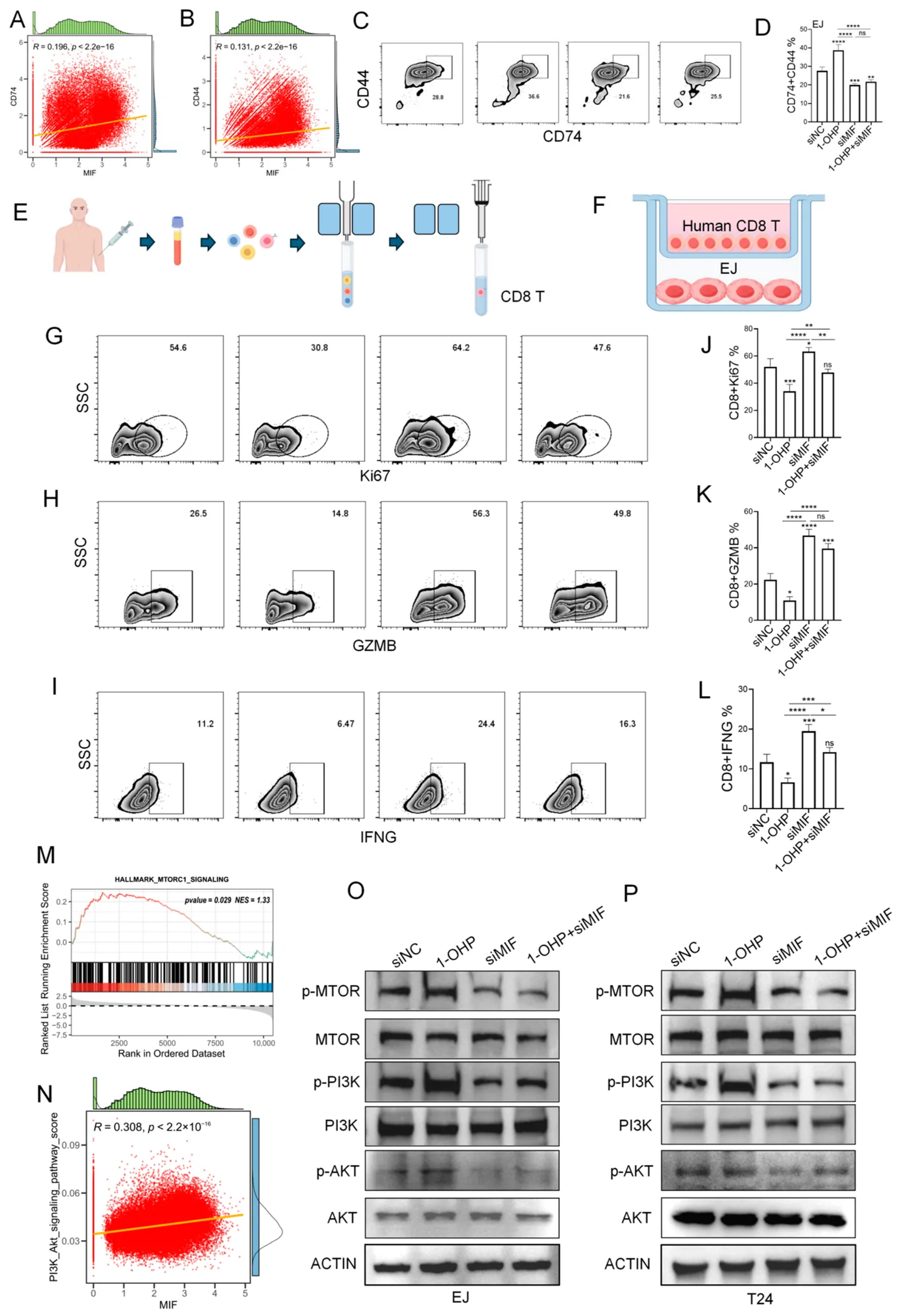
MIF mediates 1-OHP-induced tumor-associated immunosuppression and PI3K/AKT/mTOR pathway activation. (**A**,**B**) Correlation analysis showing the associations of MIF expression with CD74 and CD44, respectively. (**C**,**D**) Flow cytometric analysis of CD74 and CD44 expression in EJ cells after the indicated treatments. (**E**,**F**) Schematic illustration of PBMC isolation, CD8^+^ T-cell sorting, and co-culture with bladder cancer cells. (**G**–**L**) Flow cytometric analysis of Ki67, GZMB, and IFNG expression in CD8^+^ T cells after co-culture with differently treated EJ cells. (**M**) GSEA plot showing enrichment of the HALLMARK_MTORC1_SIGNALING pathway. (**N**) Correlation analysis showing a positive association between MIF expression and PI3K/AKT pathway activity. (**O**,**P**) Western blot analysis of PI3K/AKT/mTOR pathway activation in EJ and T24 cells under the indicated treatments. ns, not significant; * *p* < 0.05, ** *p* < 0.01, *** *p* < 0.001, **** *p* < 0.0001.

Single-cell RNA sequencing linked MIF expression with its receptors CD74 and CD44 in BCa samples (Figure 7A,B). We then measured CD74 and CD44 in EJ cells. Both proteins increased after 1-OHP treatment, and MIF knockdown reduced this increase (Figure 7C,D). The effect on CD8^+^ T cells was tested using a co-culture system. Activated CD8^+^ T cells from healthy donors were cultured with differently treated EJ cells (Figure 7E,F). Exposure to 1-OHP-treated tumor cells decreased Ki67, GZMB, and IFNG levels in CD8^+^ T cells. This decrease was weaker when MIF was knocked down in EJ cells. Ki67, GZMB, and IFNG levels were higher in the 1-OHP + siMIF group than in the 1-OHP group (Figure 7G–L). The gating strategy is provided in Figure S2. These results indicate that MIF is involved in the reduced CD8^+^ T-cell activity caused by 1-OHP-treated BCa cells. Single-cell data also linked higher MIF expression with greater PI3K/AKT and mTORC1 pathway activity (Figure 7M,N). We then examined these proteins in EJ and T24 cells. 1-OHP increased the phosphorylation of PI3K, AKT, and mTOR without clear changes in their total protein levels. MIF knockdown reduced the phosphorylation induced by 1-OHP (Figure 7O,P).

## 3. Discussion

We used bioinformatics, machine learning, molecular docking, and experimental assays to study the effects of 1-OHP in bladder cancer. MIF emerged as a main target connecting 1-OHP exposure with changes in BCa cells. 1-OHP increased cell proliferation, migration, and invasion, while MIF knockdown reduced these effects. It also increased PI3K, AKT, and mTOR phosphorylation and raised CD74/CD44 expression. These changes were weakened after MIF silencing. BCa cells treated with 1-OHP also reduced the proliferation and function of CD8^+^ T cells. This effect was partly reversed by MIF knockdown. These results place MIF between 1-OHP exposure, tumor cell behavior, and changes in CD8^+^ T-cell activity.

1-OHP is a common metabolite used to assess PAH exposure. Previous studies have linked PAH exposure with oxidative stress, inflammation, DNA damage, and cancer development. The bladder is exposed to compounds and metabolites excreted in urine, making PAH metabolites relevant to bladder carcinogenesis. The molecules that connect 1-OHP exposure with BCa progression are still not well defined. Our results identify MIF as one molecule that may take part in this process.

Six genes were selected in our computational analysis, and MIF was chosen for experimental testing. MIF is a cytokine involved in tumor growth, metastasis, inflammation, and immune regulation [18,19,20,21]. We found that 1-OHP increased MIF protein levels in BCa cells. Loss of MIF reduced the increases in proliferation, migration, and invasion caused by 1-OHP. This result suggests that MIF has a functional role in the tumor-promoting effects of 1-OHP rather than simply changing along with 1-OHP exposure.

The signaling results provide another link between MIF and the effects of 1-OHP. Treatment with 1-OHP increased the phosphorylation of PI3K, AKT, and mTOR, while MIF knockdown reduced these changes. Single-cell analysis also linked MIF with CD74 and CD44. The cell experiments gave a similar result, as the increase in CD74/CD44 after 1-OHP treatment was reduced by MIF knockdown. The MIF–CD74/CD44 axis has been linked to tumor-related signaling in previous studies. Our data suggest that 1-OHP may use this pathway to increase PI3K/AKT/mTOR activity and promote BCa progression.

The CD8^+^ T-cell results add an immune aspect to the role of MIF in 1-OHP exposure. CD8^+^ T cells cultured with 1-OHP-treated BCa cells had lower Ki67, GZMB, and IFNG levels. MIF knockdown in the tumor cells partly restored these markers. Thus, MIF released or expressed by tumor cells may be involved in the loss of CD8^+^ T-cell activity. The effects of 1-OHP may therefore involve both changes in cancer cells and altered communication with immune cells.

This study has several limitations. Most of the functional experiments were carried out in vitro. Animal studies are still needed to determine whether the 1-OHP–MIF relationship also affects tumor growth and immune responses in vivo. Molecular docking predicted possible binding between 1-OHP and the selected proteins, but it cannot prove a direct physical interaction. MIF knockdown also did not completely block the effects of 1-OHP. Other molecules or pathways may therefore take part in this process. The co-culture model also cannot reproduce all features of the tumor microenvironment in patients. Even with these limitations, our data support a role for MIF in linking 1-OHP exposure with BCa progression and immune changes. Further work is needed to test this relationship in animal models and clinical samples.

## 4. Materials and Methods

### 4.1. Acquisition of BCa-Related Transcriptomic Datasets

BCa transcriptomic data were obtained from the Gene Expression Omnibus (GEO). Four datasets were included: GSE13507, GSE32548, GSE3167, and GSE7476. GSE13507 and GSE32548 formed the discovery set. GSE3167 and GSE7476 were used for validation. Batch effects in the discovery data were assessed by surrogate variable analysis (SVA). The identified latent factors were adjusted before further analysis. Remaining batch differences were corrected with ComBat using an empirical Bayes method. Principal component analysis (PCA) was used to check the corrected data. Samples from different datasets showed better clustering after correction.

### 4.2. Identification of 1-Hydroxypyrene-Associated Targets

Information on 1-OHP, including its physicochemical properties and canonical SMILES, was obtained from PubChem and published studies. The canonical SMILES of 1-OHP was C1=CC2=C3C(=C1)C=CC4=C(C=CC(=C43)C=C2)O. ChEMBL, SwissTargetPrediction, and PharmMapper were used to search for possible protein targets of 1-OHP. The results were limited to Homo sapiens. Duplicate targets were removed before further analysis.

### 4.3. Differential Expression Analysis

Gene expression in BCa tissues was compared with that in normal bladder tissues using the limma package (version 3.68.5) in R. Genes with FDR < 0.05 and |log2FC| > 0.585 were defined as differentially expressed genes (DEGs). The expression differences were displayed using ggplot2 (version 4.0.2).

### 4.4. Weighted Gene Co-Expression Network Analysis

The WGCNA package (version 1.74) in R was used to build a gene co-expression network for BCa. Sample quality was checked with the goodSamplesGenes function. Hierarchical clustering was then carried out with a cut height of 40, and no samples were excluded. The pickSoftThreshold function was used to choose the soft-thresholding power. A β value of 6 gave a scale-free topology fit of R^2^ ≥ 0.90 while maintaining acceptable mean connectivity. The topological overlap matrix (TOM) was used to group genes into modules. Dynamic tree cutting was applied with minModuleSize = 30, deepSplit = 2, and pamRespectsDendro = FALSE. Modules with similar expression patterns were combined at a mergeCutHeight of 0.40. Pearson correlation was used to examine the association between each module and BCa. Genes with kME > 0.8 were defined as hub genes.

### 4.5. Identification of 1-OHP-Related BCa Targets and Functional Enrichment Analysis

DEGs and hub genes obtained from WGCNA were compared with the predicted targets of 1-OHP. Genes shared by these datasets were selected as candidate 1-OHP-related BCa targets and displayed in a Venn diagram. Their functions were examined with the clusterProfiler package (version 4.20.0) in R. GO analysis covered biological process (BP), cellular component (CC), and molecular function (MF). KEGG analysis was used to examine the pathways related to these genes. A *p* value < 0.05 was used as the cutoff for significance.

### 4.6. Machine Learning-Based Screening of Core Genes

Machine learning was used to screen the candidate genes related to 1-OHP and BCa. The training data were analyzed with 11 algorithms: Lasso, support vector machine (SVM), random forest (RF), glmBoost, Stepglm, Ridge, elastic net (Enet), gradient boosting machine (GBM), linear discriminant analysis (LDA), XGBoost, and Naive Bayes. These algorithms generated 113 prediction models. Five-fold cross-validation was used for parameter tuning. The data were also split into training and internal validation sets by stratified sampling. Model accuracy was assessed by the area under the receiver operating characteristic curve (AUC). Models with an AUC > 0.75 were retained. Genes appearing more often across these models were given higher priority and used to select the final core genes. Their expression levels were displayed as a heatmap.

### 4.7. Model Interpretation Using SHAP

SHapley Additive exPlanations (SHAP) was used to examine the contribution of individual genes to model predictions. A SHAP value was obtained for each gene. The values were used to assess the importance of each gene and whether it had a positive or negative effect on the model output.

### 4.8. Molecular Docking Analysis

Molecular docking was used to examine the binding between 1-OHP and the selected core targets. The SDF structure of 1-OHP was obtained from PubChem. Three-dimensional structures of the target proteins were collected from public protein structure databases. Water molecules were removed from the protein structures, and hydrogen atoms were added. The ligand geometry was optimized with the MMFF force field. Docking grids were placed around the predicted active sites. Grid size was set according to ligand size and the expected binding mode. AutoDock Vina (version 1.2.7) was used for docking. The docking results were viewed in Discovery Studio 2019.

### 4.9. Single-Cell Analysis

Single-cell RNA-seq data from GSE222315 and HRA002012 were combined for analysis. Raw UMI count matrices were loaded into Seurat. Cells with 200–6000 detected genes were kept, while cells with mitochondrial gene percentages above 20% were excluded. DoubletFinder was used to remove potential doublets. After filtering, 127,242 cells remained for further analysis. SCTransform was used for normalization and batch correction. The datasets were then integrated with FindIntegrationAnchors and IntegrateData. PCA was run on the integrated data, and the first 30 principal components were used for nearest-neighbor graph construction, clustering, and t-SNE. Cell types were assigned using canonical marker genes together with SingleR predictions and cluster-specific differentially expressed genes. The main cell populations included epithelial cells, myeloid cells, T cells, B cells, plasma cells, CAFs, endothelial cells, and mast cells. FindMarkers was used to identify differentially expressed genes. AddModuleScore was used to calculate the six-gene signature score and compare its activity among epithelial subpopulations. CellChat was used to examine ligand–receptor communication between cell types. Genes increased in MIF^+^ epithelial cells were analyzed with clusterProfiler for GO and KEGG enrichment.

### 4.10. Cell Culture and Cell Transfection

Human BCa cell lines EJ and T24 were obtained from the Cell Bank of the Chinese Academy of Sciences (Shanghai, China). Cells were grown in RPMI-1640 containing 10% fetal bovine serum (FBS) at 37 °C with 5% CO_2_. Small interfering RNAs (siRNAs) targeting MIF were synthesized. The siRNA sequences were as follows: 5′-GACAGGGTCTACATCAACTAT-3′ (siMIF-1); 5′-CTACATCAACTATTACGACAT-3′ (siMIF-2); 5′-CCTGCACAGCATCGGCAAGAT-3′ (siMIF-3).

### 4.11. Cell Proliferation, Colony Formation, and Transwell Assays

For the CCK-8 assay, 2000 cells were placed in each well of a 96-well plate. PBS was added to the outer wells to limit evaporation. Cells were treated with the indicated concentrations of 1-OHP for 24 h. The medium was then replaced with 100 μL of medium containing 10% CCK-8 reagent. After incubation at 37 °C for 2 h, absorbance was read at 450 nm. Three independent experiments were carried out. For the colony formation assay, 400 cells were placed in each well of a 6-well plate and grown for 10 days. Colonies were fixed in 4% paraformaldehyde for 30 min and stained with 0.1% crystal violet for 10 min. The plates were washed, air-dried, and photographed. For the Transwell invasion assay, cells were collected 72 h after transfection and suspended in serum-free RPMI-1640. The cell suspension was placed in the upper chamber coated with Matrigel. The lower chamber contained 500 μL of RPMI-1640 with 20% FBS. After 36 h, cells left on the upper side of the membrane were removed. Cells that passed through the membrane were fixed with 4% paraformaldehyde for 30 min and stained with 0.1% crystal violet for 10 min. The membranes were washed and photographed under a microscope. Cell proliferation was also measured with the BeyoClick™ EdU-594 Cell Proliferation Kit with AF594 (Beyotime Biotechnology, Shanghai, China) following the manufacturer’s instructions. EdU-positive cells were quantified with ImageJ (version 1.54p).

### 4.12. Western Blotting

Total protein was extracted from cells with RIPA buffer and quantified using a BCA assay. The same amount of protein from each sample was separated on 10% SDS-PAGE and transferred onto PVDF membranes. After blocking for 15 min, the membranes were kept with primary antibodies at 4 °C overnight. The antibodies included ACTIN (Abclonal, Cat. No. AC026), mTOR (Abclonal, Cat. No. A2445), p-mTOR (Abclonal, Cat. No. AP0094), AKT (Abclonal, Cat. No. A29131), p-AKT (Abclonal, Cat. No. AP1208), PI3K (Abclonal, Cat. No. A22730), and p-PI3K (Abclonal, Cat. No. AP1463). The membranes were washed with TBST and treated with the corresponding secondary antibodies (Abclonal, Cat. No. AS014) for 2 h at room temperature. Protein bands were detected with an enhanced chemiluminescence reagent. Band images were collected with a gel imaging system, and ImageJ was used to measure band intensity.

### 4.13. PBMC Isolation, CD8^+^ T-Cell Co-Culture, and Flow Cytometry

Peripheral blood was obtained from healthy volunteers after informed consent. PBMCs were separated by density gradient centrifugation. CD8^+^ T cells were isolated from PBMCs with magnetic beads following the manufacturer’s protocol. The cells were activated with CD3/CD28 antibodies, and IL-2 was added to the culture medium. Activated CD8^+^ T cells were then cultured with treated bladder cancer cells at a 5:1 ratio for 48 h. After co-culture, the cells were harvested and washed with cold PBS. Dead cells were excluded with FVS780. CD8 surface staining was carried out for 45 min at 4 °C in the dark. The cells were then fixed and permeabilized. Ki67, GZMB, and IFNG were stained intracellularly for 45 min at 4 °C in the dark. After washing, the cells were suspended in PBS for flow cytometric analysis. For CD74 and CD44 detection, treated tumor cells were harvested and washed twice with cold PBS. FVS780 was used to exclude dead cells. Fluorochrome-conjugated antibodies against CD74 and CD44 were added for 45 min at 4 °C in the dark. The cells were washed, suspended in PBS, and examined by flow cytometry.

### 4.14. Statistical Analysis

Differences between two groups were tested with a two-tailed Student’s *t*-test. One-way or two-way ANOVA was applied when more than two groups were compared. Tukey’s test was used for post hoc comparisons when needed. Statistical significance was set at *p* < 0.05. Single-cell RNA-seq data were analyzed in R (version 4.4.1). The analysis covered data integration, cell clustering, differential expression, module scoring, and cell–cell communication.

## 5. Conclusions

Our results identify MIF as a mediator of the effects of 1-OHP in bladder cancer. 1-OHP increased BCa cell proliferation, migration, and invasion and raised MIF expression. It also increased CD74/CD44 expression and PI3K/AKT/mTOR activity. In the co-culture model, BCa cells treated with 1-OHP reduced CD8^+^ T-cell proliferation and function, while MIF knockdown partly reduced these effects. These results link MIF to both the tumor-promoting and immune effects of 1-OHP. Studies in animal models and clinical samples are needed to confirm the role of the 1-OHP–MIF axis and determine its relevance in bladder cancer.

## Data Availability

The original contributions presented in the study are included in the article. Further inquiries can be directed to the corresponding author.

## Supplementary Materials

The following supporting information can be downloaded at: https://www.mdpi.com/article/10.3390/ijms27177778/s1.

## References

[1] Jubber I., Ong S., Bukavina L., Black P.C., Comperat E., Kamat A.M., Kiemeney L., Lawrentschuk N., Lerner S.P., Meeks J.J. (2023). Epidemiology of Bladder Cancer in 2023: A Systematic Review of Risk Factors. Eur. Urol..

[2] Thangphatthanarungruang J., Chotsuwan C., Siangproh W. (2023). A novel and easy-to-construct polymeric l-glutamic acid-modified sensor for urinary 1-hydroxypyrene detection: Human biomonitoring of polycyclic aromatic hydrocarbons exposure. Talanta.

[3] St Helen G., Goniewicz M.L., Dempsey D., Wilson M., Jacob P., Benowitz N.L. (2012). Exposure and kinetics of polycyclic aromatic hydrocarbons (PAHs) in cigarette smokers. Chem. Res. Toxicol..

[4] Clauzel A., Persoons R., Maitre A., Balducci F., Petit P. (2024). Review of environmental airborne pyrene/benzo[a]pyrene levels from industrial emissions for the improvement of 1-hydroxypyrene biomonitoring interpretation. J. Toxicol. Environ. Health B Crit. Rev..

[5] Feng L., Huang G., Peng L., Liang R., Deng D., Zhang S., Li G., Wu S. (2024). Comparison of bladder carcinogenesis biomarkers in the urine of traditional cigarette users and e-cigarette users. Front. Public Health.

[6] Valiere M., Petit P., Persoons R., Demeilliers C., Maitre A. (2022). Consistency between air and biological monitoring for assessing polycyclic aromatic hydrocarbon exposure and cancer risk of workers. Environ. Res..

[7] Adly H.M., Saleh S.A. (2022). The Association of Increased Oxidative Stress and Tumor Biomarkers Related to Polyaromatic Hydrocarbons Exposure for Different Occupational Workers in Makkah, Saudi Arabia. Cureus.

[8] Chen C.S., Yuan T.H., Lu T.P., Lee H.Y., Chen Y.H., Lai L.C., Tsai M.H., Chuang E.Y., Chan C.C. (2024). Exposure-associated DNA methylation among people exposed to multiple industrial pollutants. Clin. Epigenet..

[9] van Hoogstraten L.M.C., Vrieling A., van der Heijden A.G., Kogevinas M., Richters A., Kiemeney L.A. (2023). Global trends in the epidemiology of bladder cancer: Challenges for public health and clinical practice. Nat. Rev. Clin. Oncol..

[10] Filho A.M., Briganti A., Jemal A., Bray F. (2025). Bladder Cancer Incidence and Mortality: A Global Overview and Recent Trends. Eur. Urol..

[11] Guo L., Zhang X., Li X., Wang K., Wang Y., Abulikemu A., Su X., Shu M., Li H., Cui S. (2024). Polycyclic aromatic hydrocarbon and its adducts in peripheral blood: Gene and environment interaction among Chinese population. Environ. Int..

[12] Olsson A., Bouaoun L., Schuz J., Vermeulen R., Behrens T., Ge C., Kromhout H., Siemiatycki J., Gustavsson P., Boffetta P. (2024). Lung Cancer Risks Associated with Occupational Exposure to Pairs of Five Lung Carcinogens: Results from a Pooled Analysis of Case-Control Studies (SYNERGY). Environ. Health Perspect..

[13] Zhou S., Zhu Q., Liu H., Jiang S., Zhang X., Peng C., Yang G., Li J., Cheng L., Zhong R. (2021). Associations of polycyclic aromatic hydrocarbons exposure and its interaction with XRCC1 genetic polymorphism with lung cancer: A case-control study. Environ. Pollut..

[14] Weng M.W., Lee H.W., Park S.H., Hu Y., Wang H.T., Chen L.C., Rom W.N., Huang W.C., Lepor H., Wu X.R. (2018). Aldehydes are the predominant forces inducing DNA damage and inhibiting DNA repair in tobacco smoke carcinogenesis. Proc. Natl. Acad. Sci. USA.

[15] Roos P.H., Bolt H.M. (2005). Cytochrome P450 interactions in human cancers: New aspects considering CYP1B1. Expert. Opin. Drug Metab. Toxicol..

[16] Dyrskjot L., Hansel D.E., Efstathiou J.A., Knowles M.A., Galsky M.D., Teoh J., Theodorescu D. (2023). Bladder cancer. Nat. Rev. Dis. Primers.

[17] Hurst C.D., Alder O., Platt F.M., Droop A., Stead L.F., Burns J.E., Burghel G.J., Jain S., Klimczak L.J., Lindsay H. (2017). Genomic Subtypes of Non-invasive Bladder Cancer with Distinct Metabolic Profile and Female Gender Bias in KDM6A Mutation Frequency. Cancer Cell.

[18] Song J., Song X., Xie Y., Guo H., Cun Y. (2026). MIF-expressing tumor cells mediate immunotherapeutic resistance in esophageal squamous cell carcinoma. Theranostics.

[19] Chen W., Liao Y., Yao H., Zou Y., Fang J., Zhang J., Guo Z., Tu J., Chen J., Huo Z. (2025). Multiomics integration analysis identifies tumor cell-derived MIF as a therapeutic target and potentiates anti-PD-1 therapy in osteosarcoma. J. Immunother. Cancer.

[20] Xu T., Zhao W., Zha W., Zhang J., Zeng F., Wu D., Miao C., Zhang H. (2026). The functional roles of macrophage migration inhibitory factor in the tumor microenvironment. Cytokine Growth Factor. Rev..

[21] Altulea A., Nehme J., Demaria M. (2026). Dual role of MIF in aging and cellular senescence. Cytokine Growth Factor. Rev..

