# Supplementary material for: 1-Hydroxypyrene Promotes Bladder Cancer Progression and Tumor-Associated Immunosuppression via MIF
Source: Int J Mol Sci. 2026 Aug 30;27(17):7778. doi: 10.3390/ijms27177778 (PMC13566501; doi:10.3390/ijms27177778)
Supplement: Supplementary file 1 [file ijms-27-07778-s001.zip › Figure S1+Figure S2.pdf]

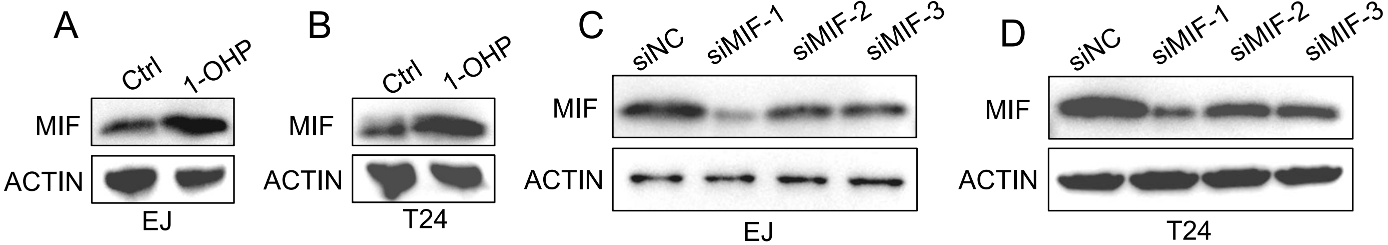
A
Ctrl
1-OHP
MIF
ACTIN
EJ
B
Ctrl
1-OHP
MIF
ACTIN
T24
C
siNC
siMIF-1
siMIF-2
siMIF-3
MIF
ACTIN
EJ
D
siNC
siMIF-1
siMIF-2
siMIF-3
MIF
ACTIN
T24

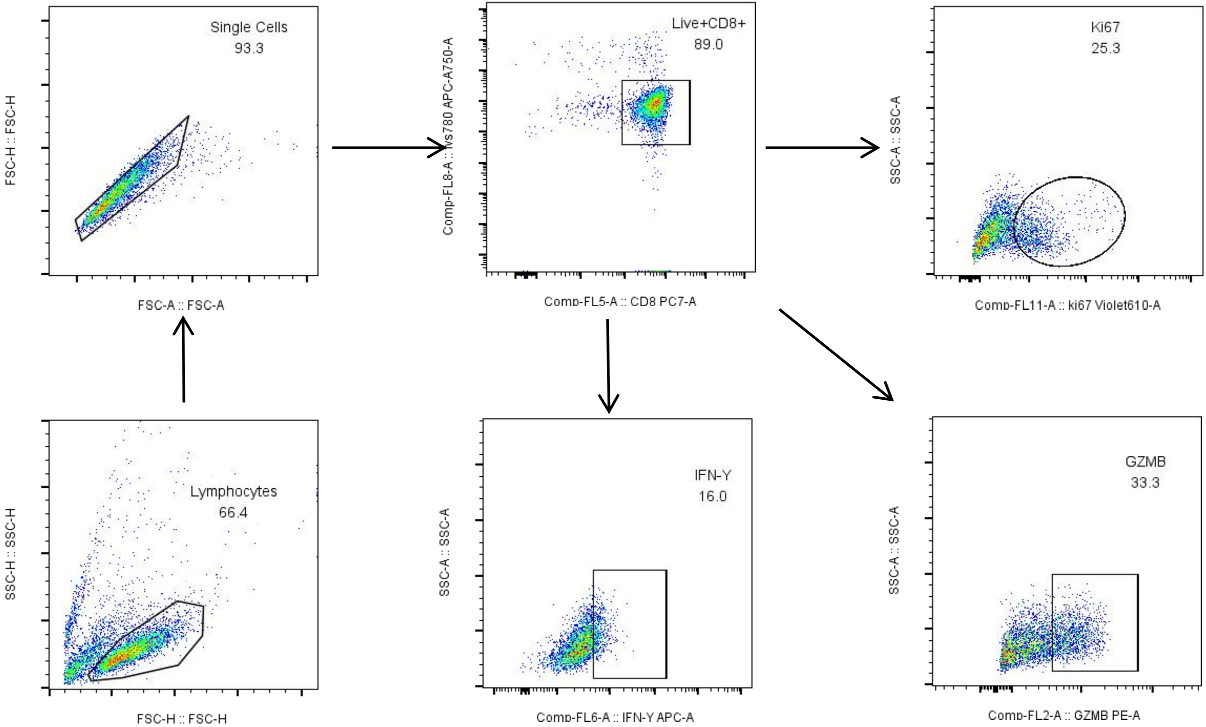

Single Cells
93.3
FSC-H :: FSC-H
FSC-A :: FSC-A
Live+CD8+
89.0
Comp-FL8-A :: lvs780 APC-A750-A
Comp-FL5-A :: CD8 PC7-A
Ki67
25.3
SSC-A :: SSC-A
Comp-FL11-A :: ki67 Violet610-A
Lymphocytes
66.4
SSC-H :: SSC-H
FSC-H :: FSC-H
IFN-Y
16.0
SSC-A :: SSC-A
Comp-FL6-A :: IFN-Y APC-A
GZMB
33.3
SSC-A :: SSC-A
Comp-FL2-A :: GZMB PE-A
