# Supplementary material for: 1-Hydroxypyrene Promotes Bladder Cancer Progression and Tumor-Associated Immunosuppression via MIF
Source: Int J Mol Sci. 2026 Aug 30;27(17):7778. doi: 10.3390/ijms27177778 (PMC13566501; doi:10.3390/ijms27177778)
Supplement: Supplementary file 1 [file ijms-27-07778-s001.zip › Supplementary Materials.pdf]

Legend of Figure:

Figure S1. Validation of 1-OHP-induced MIF expression and siRNA-mediated MIF knockdown in bladder cancer cells.

(A–B) Western blot analysis of MIF expression in EJ and T24 cells after 1-OHP treatment.

(C–D) Western blot verification of MIF knockdown efficiency in EJ and T24 cells transfected with three independent siRNAs.

Figure S2. Flow cytometric gating strategy for functional analysis of $CD8^+$ T cells

Gating strategy for flow cytometric analysis of live $CD8^+$ T cells.
